# A possible systemic rheumatic disorder in the Nova Scotia duck tolling retriever

**DOI:** 10.1186/1751-0147-51-16

**Published:** 2009-03-30

**Authors:** Helene Hansson-Hamlin, Inger Lilliehöök

**Affiliations:** 1 The Department of Clinical Sciences, The Swedish University of Agricultural Sciences, Uppsala, Sweden; 2 The University Animal Hospital, The Swedish University of Agricultural Sciences, Uppsala, Sweden

## Abstract

**Background:**

A disease complex with chronic musculoskeletal signs, including stiffness and joint pain, and to which there is a strong predisposition in the canine breed Nova Scotia duck tolling retriever (Toller) has been recognized in Sweden. The aim of this first clinical description of the disorder in Tollers was to describe the clinical manifestations and laboratory findings, as well as to try to identify a possible immune-mediated background of the disease and to show the outcome of treatment in 33 Tollers.

**Methods:**

The study included 33 Tollers with musculoskeletal signs and 20 healthy controls. All the dogs were thoroughly examined and followed for a period of 2 months – 4 years. An IIF-ANA (antinuclear antibody) test and an assay for the presence of antibodies to *Anaplasma phagocytophilum *and *Borrelia burgdorferi *sensu lato were performed, as well as some haematology, serum biochemistry and urine tests. Routine radiographic examinations were performed on 11 dogs.

**Results:**

All the Toller patients showed stiffness and lameness that had lasted for at least 14 days and displayed pain from several joints of extremities on manipulation. Twenty-seven per cent of the dogs also showed muscle pain and 18% different skin symptoms. Seventy per cent of the Tollers with signs of disease displayed a positive IIF-ANA test. Most of the dogs were treated with corticosteroids, with the majority of the dogs (65%) showing good responses. There was no association between the IIF-ANA results and the clinical signs or results of treatment.

**Conclusion:**

This paper describes a disorder in Nova Scotia duck tolling retrievers where the clinical signs, ANA reactivity and response to corticosteroids strongly suggest that the disorder is immune-mediated. The findings of this research may indicate a chronic systemic rheumatic disorder.

## Background

In recent years a disease involving chronic musculoskeletal signs with stiffness and pain from several joints has been recognized in Sweden in the canine breed Nova Scotia duck tolling retriever (Toller). Other concomitant findings, such as fever and skin problems, are unusual but may be apparent. Often the symptoms resemble those seen in systemic autoimmune rheumatic diseases. In addition, other immune-mediated conditions, such as Addison's disease and aseptic meningitis (also called steroid-responsive meningitis-arteritis, SRMA), have been reported to occur at a high frequency in the Toller breed [1-3].

In human medicine, rheumatic diseases are autoimmune disorders where the clinical problems involve joints, soft tissues and allied conditions of connective tissues. Systemic autoimmune diseases in dogs have previously mainly been referred to as systemic lupus erythematosus (SLE). One hallmark of SLE is high titres of circulating antinuclear antibodies (ANA), which can be demonstrated by the indirect immunofluorescence (IIF) ANA test. Several efforts have been made to identify definite criteria for SLE in the dog, as has been attempted in the case of human SLE [4,5], but no uniform list of such criteria for dogs has so far been presented. Clinical signs that have been described are, e.g., musculoskeletal disorders, skin disorders, anaemia, thrombocytopenia, polymyositis, nephropathy and fever [6-13].

Besides SLE, other systemic ANA-positive autoimmune diseases, referred to as SLE-related diseases, have been described in human patients, in many cases with overlapping diagnostic features. More recently, it has also been suggested that SLE-related diseases affect dogs. However, these diseases have shown overlapping clinical signs, as has been described for human patients [2,14-16].

Although uncommon, immune-mediated myositis in the dog has also been described [17,18]. In Sweden a large proportion of dogs are registered in the Swedish Kennel Club (SKC), with pedigree information for each individual. There is, in comparison with other countries, a large population of Tollers in Sweden, with approximately 3,200 dogs according to data from SKC and the Nova Scotia Duck Tolling Retriever Club of Sweden. These conditions make studies of diseases in this breed in Sweden unique. According to the Swedish Kennel Club, newly registered Tollers constitute approximately 0.6% of the newly registered canine population in Sweden.

The purpose of this study was to describe the clinical findings in 33 Tollers with a chronic musculoskeletal disorder and to try to identify a possible immune-mediated background of the disease. The investigations included the history, clinical signs, antinuclear antibody (ANA) reactivity, haematological, serum biochemical and radiological findings, and the treatment and progress of the disease and were compared to results from 20 healthy Tollers.

## Methods

### Patients

The Nova Scotia duck tolling retrievers (Tollers) in this study, n = 33, were privately owned and first examined at the University Animal Hospital at the Swedish University of Agricultural Sciences (SUAS) in Uppsala during January 2002 – January 2007. The clinical examination included a thorough palpation and manipulation of the joints, back and neck. All the Tollers with musculoskeletal signs indicating a systemic rheumatic disorder, including stiffness and pain from at least two different joints of extremities, were included in the study. These signs were to have been apparent for at least 14 days and were the main reason for the dog owner to visit the University Animal Hospital. No other disease was suspected by the veterinary surgeon as the main cause of the signs shown. However, one Toller patient with clinical findings that fulfilled the inclusion criteria was found later on to have acute myeloid neoplasia, and consequently the dog was excluded from the study.

Twenty-seven (82%) of the dogs were not receiving treatment when they were included in the study. Five of the dogs were receiving treatment with prednisolone, the dosage varying between 0.15 mg/kg and 1 mg/kg every second day, and one dog was on NSAID treatment (carprofen 4 mg/kg and day). These six dogs had been receiving treatment for at least 14 days at the time of the blood sampling.

All the dogs were followed during a period of 2 months – 4 years depending on the time for entrance into the project.

The study was approved by the local ethical committee (C79/7).

### Healthy controls

Twenty apparently healthy privately owned Tollers, examined at the University Animal Hospital at SUAS in Uppsala, were included in the study and had no clinical signs of disease. The control dogs did not show any signs of disease, either at the initial appointment, or at the follow-up contacts, which took place between 4 months and 3 years later. Among the 20 healthy control dogs, there were10 males, three of which were castrated, and 10 females, two of which were spayed. The age distribution was 9 months – 11 years, with a median age of 5.5 years.

### IIF-ANA tests

The IIF-ANA tests were performed using monolayers of HEp-2 cells fixed on glass slides (Immuno Concepts, Sacramento, CA, USA), as described previously [19]. The slides were examined by fluorescence microscopy and considered positive at the dilution ≥ 1/100. The nuclear fluorescence pattern was used to subdivide the results into homogeneous and speckled patterns, as previously described [2,19-21].

From 30 of the 33 Toller patients, a new serum sample was obtained for IIF-ANA analysis 2 – 4 months later.

### Haematology and serum biochemistry tests, and urine analysis

Blood samples were drawn into EDTA and serum clot tubes on the first day of admission to the hospital.

The haemoglobin concentration (diseased dogs n = 29/33 and controls n = 20/20) and total WBC counts (n = 30/33 and 20/20) were analyzed using a Cell-Dyn 3500 analyzer (Abbott Diagnostics, Santa Clara, CA, US), with leukocyte differential counts obtained automatically, together with a manual examination of the blood smear and, if needed, a manual differential count.

The fibrinogen (n = 24/33 and 18/20), bile acids (n = 30/33 and 20/20), creatinine (n = 32/33 and 20/20), aspartate aminotransferase (AST) (n = 31/33 and 20/20) and total protein concentration (n = 32/33 and 20/20) were analysed using an auto analyzer (Konelab 30, Thermo Electron Corporation, Vantaa, Finland). Commercial reagents from Thermo were used for the creatinine, AST and total protein concentration. The fibrinogen was analysed with a reagent from Kamiya Biomedical Company (Seattle, US) and the bile acids with reagents from Diazyme (Hannover, Germany).

Serum protein electrophoresis (n = 31/33 and 20/20) was performed on an agarose gel system (Sebia Hydrasys LC) according to standard procedures. The protein pattern was divided into six fractions, albumin and α_1_-, α_2_-, β_1_-, β_2_- and γ-globulins, using a laser densitometer (Sebia Hyrys2, Sebia, Issy-les-Molineaux, France).

Urine dipsticks (Combur-Test, Roche Diagnostics GMbH, Mannheim, Germany) were used for analysis of the pH, haemoglobin, glucose and ketone. The specific gravity was determined with a refractometer, the protein was measured with a sulfosalicylic acid precipitation test and the urine sediment was examined microscopically. Urine analysis was performed for 27 dogs (17 patients and 10 controls).

### Serology and radiology examinations

Serum samples from 32 sick Tollers and from all the healthy controls were analyzed by an immunofluorescence assay for the presence of antibodies to *Anaplasma phagocytophilum *[22,23] and *Borrelia burgdorferi *sensu lato (a Swedish isolate of *B Afzelii *[24]). The tests were considered positive at a titre of ≥ 1:80 (Borrelia) and ≥ 1:40 (Anaplasma).

Routine radiologic examinations of painful joints were performed on 11 patients.

### Statistics

The Mann-Whitney test (Minitab Inc, Coventry, UK) was used to compare laboratory results between the diseased and healthy Tollers.

## Results

### Age and gender

Seventeen (52%) of the Toller patients were males, two of which were castrated, and 16 (48%) were females, one of which was spayed. The age distribution was 10 months–11 years, with a median age of 3 years. However, there was only one dog aged 11 and one dog aged 9, and the other dogs were all ≤ 7 years of age (Table 1). The two older dogs had shown clinical signs for a long period of time, according to the owners, before entrance into the study.

**Table 1:** Age, gender, clinical signs, and haematology, biochemistry and ANA results for each of the Nova Scotia duck tolling retrievers with a possible systemic rheumatic disorder.

**Dog no**	**Age (years)/gender**	**Clinical signs**	**Haematology**	**Biochemistry**	**ANA**	**Analysis not done**
1	5/M	st, jp, mp	WBC↓, neutr.↓, lymph.↓		H	fibr
2	3/M	st, jp, skin			H	
3	2/F	st, jp, mp	lymph.↓		Sp.	
4	3/F	st, jp, skin		Alb.↓	H	
5	11/F	st, jp		Prot.↑, γ↑	H	
6	5/F	st, jp		γ↑	Sp.	
7	9/M	st, jp		Prot.↓, Alb.↓, α2↑, AST↑	Neg.	fibr
8	7/M	st, jp		β2↑, γ↑	Neg.	
9	2/F	st, jp		γ↑, AST↑, b.a.↑	Sp.	
10	5/M	st, jp	WBC↓, neutr.↓, lymph.↓		H	
11	1/F	st, jp, mp			Sp.	
12	1/F	st, jp, mp			Sp.	
13	3/M	st, jp		Alb.↓, β2↑, γ↑, AST↑	H	
14	4/M	st, jp, mp		α2↑	Sp.	Hb, WBC, fibr
15	3/F	st, jp, f		Prot.↑, β1↑, β2↑, γ↑	Sp.	fibr
16	5/M	st, jp, skin		γ↑	Neg.	fibr
17	2/M	st, jp			Neg.	
18	2/M	st, jp, skin			Neg.	
19	4/F	st, jp, mp	lymph.↓		Neg.	
20	6/F	st, jp	WBC↓, lymph.↓		Neg.	
21	1/F	st, jp	Hb↓		Sp.	
22	2/M	st, jp			Neg.	fibr, b.a.
23	10 mon./M	st, jp, mp, f			Neg.	fibr
24	3/F	st, jp, skin			H	Haematol. and biochem.
25	5/F	st, jp		α2↑, β1↑	H	
26	1/M	st, jp, f			Sp.	
27	4/F	st, jp, skin	WBC↑	α2↑, γ↑, b.a.↑	H	
28	4/F	st, jp		β2↑, γ↑, AST↑	Sp.	
29	1/F	st, jp, mp		AST↑	Neg.	
30	1/M	st, jp			Sp.	
31	2/M	st, jp			Sp.	
32	2/M	st, jp, mp		β2↑	Sp.	Hb, WBC, fibr
33	1/M	st, jp, f	neutr.↓		Sp.	Hb, AST, b.a., el.phoresis

Only haematology and biochemistry values outside the reference values are demonstrated.

#M = male, F = female, mon. = months

st = stiffness, jp = joint pain, mp = muscle pain, skin = skin problems, f. = fever

neutr = neutrophils, lymph = lymphocytes, b.a. = bile acids, fibr = fibrinogen

Prot. = protein, Alb. = albumin, α2 = α2-globulins, β1 = β1-globulins, β2 = β2-globulins, γ = γ-globulins

↑ = value higher than reference value, ↓ = value lower than reference value

H = homogeneous staining, Sp. = speckled staining, Neg. = negative

### Clinical findings

Each Toller patient suffered from a persistent lameness, with the joint pain waxing and waning, affecting different legs and joints. The joints most often affected were carpal joints, elbows and knees. Moreover, all the dogs displayed stiffness, mainly after rest, which became less apparent after exercise (Table 1, 2). None of the dogs showed any signs of depression or anorexia. For fourteen of the dogs, the duration of the signs before inclusion in the study had been 2 weeks to 2 months, for nine of the dogs 3–6 months, for five of the dogs between 7 and 12 months, and for the remaining 5 dogs more than one year. According to the owners, 85% of the dogs showed initial signs of disease at an age between 1 and 5 years.

**Table 2:** Clinical findings in Nova Scotia duck tolling retrievers with a systemic rheumatic disorder.

**Clinical signs**	**Number of dogs**
Stiffness	33 (100%)
Pain from ≥ 2 joints of extremities	33 (100%)
Muscle pain	9 (27%)
Skin disorder	6 (18%)
Fever	4 (12%)

At the clinical examination, all the Toller patients displayed pain from 2 or more joints of extremities on manipulation, with 11 of them showing pain from ≥ 4 joints on this occasion. However, none of the dogs showed any joint swelling, even though specific joints were painful when manipulated. Moreover, a total of 9 dogs also showed pain when muscles were palpated, with 6 of them having the muscle pain diffusely distributed and 3 of them displaying more pronounced local muscle pain. Two of these 3 dogs showed pain from the masseter muscles and one of them showed pain from muscles of both hind legs. None of the dogs showed signs of neck pain. An elevated body temperature was found in 4 dogs (39.5–40.4°C). Although other concomitant signs of disease were unusual, 6 of the dogs showed different skin symptoms (Table 1, 2). One of these dogs showed vitiligo and one dog displayed swelling of the nose (the skin biopsy result indicating "plasmacytic and pyogranulomatous dermatitis with occasional panniculitis"). The remaining 4 dogs showed unspecific crusting lesions on the bridge of the nose and on the ear pinnae, one of the dogs also had ulcers of the gingiva (Table 1, 2). Another dog had previously (before entrance into the study) also been diagnosed as having hypothyroidism and was receiving thyroxin treatment.

### ANA determination

**Figure 1:**
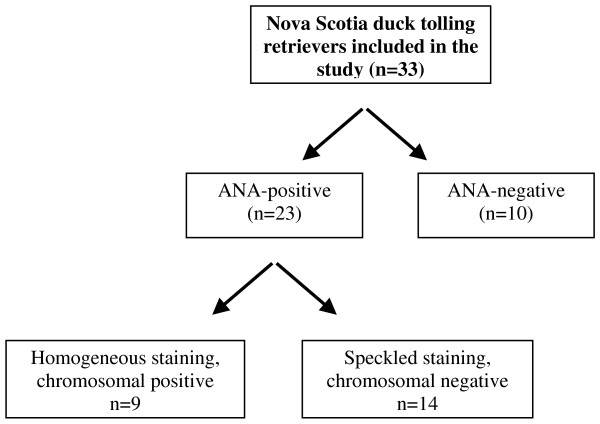
**Flow chart for indirect immunofluorescence-antinuclear antibody IIF-ANA-positive sera from Nova Scotia duck tolling retrievers with a systemic rheumatic disorder**.

Twenty-three of the Tollers with signs of disease (i.e. 70%) displayed a positive IIF-ANA test, while the remaining 10 dogs showed a negative result (Fig 1, Table 1).

A homogeneous IIF-ANA staining pattern was displayed by 39% (9/23) of the ANA-positive dog sera, all of which concomitantly displayed reactivity towards chromosomal regions in mitotic cells. A speckled IIF-ANA fluorescence pattern was shown by 61% (14/23) of the dog sera, all of which had concomitant absence of chromosomal reactivity (Fig 1, Table 1). The ANA test was repeated after two-three months, with the same positive result and the same IIF-ANA pattern.

When comparing the ANA results and different clinical signs, there were no symptoms that were clearly over-represented according to ANA positivity/negativity. One observation, however, was that 4 out of 6 dogs with skin changes were ANA-positive, all displaying homogeneous fluorescence patterns.

The sera from all the healthy controls were negative in the ANA test.

### Haematology and serum biochemistry tests, and urine analysis

The acute phase markers fibrinogen and alpha-2 globulin were significantly higher in Tollers with symptoms in comparison with the 20 Tollers without symptoms, while the lymphocytes were significantly lower (Table 3). If the 5 dogs on glucocorticoid treatment were excluded, the diseased Tollers still had significantly higher fibrinogen values (p = 0.002) and lowered lymphocyte counts (p = 0.006). The other parameters did not show any significant difference.

**Table 3:** Haematology and biochemistry results from 33 diseased Tollers and 20 control Toller dogs.

**Analysis**	Reference Interval*	**Toller patients**		**Healthy control Tollers**		
		Mean value*	**No of dogs outside ref. values (%)**	Mean value*	**No of dogs outside ref. values (%)**	**P value**
Haemoglobin (29/20)	132–199 g/L	156.5	1 (3)	155.4	2 (10)	ns
WBC (30/20)	5.2–14.1 × 10^9^/L	7.8	4 (13)	8.5	1 (5)	ns
Neutrophils (30/20)	2.8–10.4 × 10^9^/L	4.9	3 (12)	5.6	0	ns
Eosinophils (30/20)	0.1–1.3 × 10^9^/L	0.5	0	0.5	1 (5)	ns
Lymphocytes (30/20)	0.9–4.5 × 10^9^/L	1.4	5 (19)	1.9	1 (5)	0.004
Monocytes (30/20)	0.1–1.5 × 10^9^/L	0.5	0	0.5	0	ns
						
Creatinine (32/20)	40–130 μmol/L	81.1	0	81.4	0	ns
Fibrinogen (24/18)	0.7–4.5 g/L	2.7	0	1.9	0	0.001
AST (31/20)	< 0.7 μkat/L	0.6/0.4	5 (16)	0.4	0	ns
Bile acids (30/20)	< 30 μmol/L	8.3/3.2	2 (7)	7.9/5.9	1 (5)	ns
						
Total protein (31/20)	53–74 g/L	64.4	3 (9)	62.4	1 (5)	ns
Albumin (31/20)	26–41 g/L	29.3	3(10)	30.0	2 (10)	ns
α1-globulins (31/20)	1–3 g/L	2.0	0	2.0	0	ns
α2-globulins (31/20)	7–14 g/L	12.1	5 (16)	10.8	0	0.02
β1-globulins (31/20)	3–8 g/L	4.0	1 (3)	3.7	0	ns
β2-globulins (31/20)	5–11 g/L	9.4	6 (19)	9.3	2 (10)	ns
γ-globulins (31/20)	3–9 g/L	8.0/7.0	10 (32)	6.7/6.0	4 (20)	ns

The number of samples analysed for each analysis is written within brackets (diseased dogs/control dogs) in the first column.

ns = not significant

* Both mean and median results (mean/median) are presented if they did not agree

One of the Toller patients showed a mild thrombocytopenia (102 × 10^9^) with a concomitant leucopoenia (1.7 × 10^9^) and lymphocytopenia (0.4 × 10^9^). This dog displayed a homogeneous IIF-ANA pattern (Table 1). Five of the Toller patients demonstrated AST values above the reference range, with only one of them showing clinical signs of myositis. Three of these dogs were ANA-positive and 2 were ANA-negative (Table 1). Urine analysis was performed for 27 dogs (17 patients and 10 controls), and all the results were considered unremarkable. One ANA-negative canine patient had severe hypoalbuminaemia (9 g/L). The urine of this dog was not examined. Some Tollers in both groups had γ- and/or β_2_-globulins above the reference values, but no difference was observed between the two groups.

### Anaplasma phagocytophilum and Borrelia burgdorferi sensu lato

Serum samples from 32 dogs with signs of disease and from all the 20 control dogs were analysed for the presence of antibodies to *Anaplasma phagocytophilum *and *Borrelia burgdorferi *sensu lato.

The sera from the dogs with signs of disease: Three of the sera displayed a positive titre for *Anaplasma phagocytophilum *(1:160 n = 2, 1:640 n = 1). Two of these dogs had been treated with doxycycline during 3–6 weeks before admittance, but with no improvement of the clinical signs. The third dog (1:160) was not treated with antibiotics at all. One of the dogs tested positive for *Borrelia burgdorferi *(1:80). This dog was treated with amoxicillin with no improvement of the clinical signs.

The sera from the healthy control dogs: Five of the sera displayed a positive titre for *Anaplasma phagocytophilum *(1:40 n = 1, 1:160 n = 4), and 5 of the sera were positive for *Borrelia burgdorferi *(1:80 n = 1, 1:160 n = 1, 1:320 n = 2, ≥ 1:640 n = 1). Two of these dogs displayed titres for both *Anaplasma phagocytophilum *and *Borrelia burgdorferi*. None of the healthy control dogs showed any signs of disease and therefore were not treated in any way.

### Radiographic findings

In 11 of the Toller patients, painful joints were radiographically examined, and all of them were considered radiographically normal. These 11 dogs included two out of three of the oldest dogs in the study (dog no 5 and 8, Table 1).

### Treatment and prognosis

Treatment with corticosteroids (prednisolone) was the most common treatment and was used for 25 dogs. In 10 of these 25 cases an NSAID was tried initially and was later replaced by the corticosteroid treatment (Table 4). The initial period of NSAID treatment varied from 1–6 months. The remaining eight dogs were treated with only an NSAID (Table 4). All the dogs were followed during a period between 2 months and 4 years, depending on the time for entrance into the project.

**Table 4:** Response to treatment with an NSAID and/or corticosteroids in 33 Nova Scotia duck tolling retrievers with a possible systemic rheumatic disorder.

**Treatment**	**No of dogs**	**Duration**	**Outcome**
Corticosteroids	15	2 months – 2 years	Rem. clin. signs, withdrawn treatment: 5 Rem. clin. signs with treatment low dose: 3 Rem. clin. signs with treatment high dose: 3 Euthanized (no improvement despite high dose): 2 Unknown: 1
			
NSAID replaced by corticosteroids	10	2 months – 4 years	Rem. clin. signs, withdrawn treatment: 1 Rem. clin. signs with treatment corticosteroids low dose: 7 Euthanized/died (no improvement despite high dose): 3
			
NSAID only	8	2 months – several periods during 1 year	All reported minimal-moderate improvement. Euthanized: 2

Rem. clin. signs = Remission of clinical signs

The Tollers treated with corticosteroids received an initial dose of 1–1.5 mg/kg/day, which was gradually lowered during several months. Sixteen out of 25 dogs showed during treatment with a low dose (≤ 0.5 mg/kg every 2 days) almost complete remission of the symptoms, and 6 of these dogs could eventually manage without any medication at all. A higher dose (≥ 0.5 mg/kg/day) was required by 3/25 dogs to obtain enough remission of the clinical signs and 5/25 dogs (dog no 2, 5, 6, 11 and 16, Table 1) were euthanized because of bad response (insufficient remission of the symptoms) to treatment (Table 4). In one dog (the dog with hypoalbuminaemia) there was no improvement after treatment with corticosteroids and the dog died three months later.

Another dog showed almost no signs of disease during 4–5 months, but then its condition deteriorated again despite an increase in the corticosteroid dose and the dog was then euthanized. The effect of corticosteroid treatment was unknown for one dog.

All the dogs treated only with an NSAID showed minimal-moderate improvement. Of the 8 dogs treated with only an NSAID, 2 dogs (dog no 10 and 18, Table 1) were euthanized because of the bad response to treatment.

There was no association when comparing different ANA results and the results of treatment.

### Pedigree information

Five of the Tollers with signs of disease were litter mates (dog no 11, 12, 21, 31 and 32, Table 1) and one other dog had the same father as these five dogs (no 33, Table 1). These 6 dogs all entered the study at 1–2 years of age, showed similar clinical signs and displayed a speckled IIF-ANA staining pattern. Two other Tollers with signs of disease were closely related (a father and daughter) and they showed different IIF-ANA staining patterns (homogeneous and speckled).

No other dogs (neither Tollers with signs of disease nor healthy controls) were close relatives, i.e. to the level of grandparents.

## Discussion

During five years, 33 Nova Scotia duck tolling retrievers with a disease complex with prolonged stiffness and joint pain from different legs were examined at the University Animal Hospital in Uppsala. This clinical picture in Tollers is well known among breeders and veterinary surgeons in Sweden. Stiffness, mainly after rest, and chronic pain from several joints of extremities, in some cases accompanied with muscle pain, were the most common findings. In order to describe this disease in Tollers further and to exclude other chronic diseases, haematology, serum biochemistry and serology tests, urine analysis, radiographic examinations and ANA (antinuclear antibody) tests were performed. The majority of the dogs displayed ANA reactivity and the symptoms usually decreased with corticosteroid treatment. The clinical signs, the ANA reactivity and the response to corticosteroids strongly suggest that the disorder described in this study in the Toller breed is immune-mediated and the findings may indicate a chronic systemic rheumatic disorder [6,14,25].

The dogs in this study did not show signs of neck pain, depression or anorexia, which is in contrast to the findings in Tollers suffering from SRMA [3]. Moreover, Tollers suffering from SRMA are usually affected at a young age (4–19 months), while the Toller patients with the musculoskeletal disorder in our study were affected at a median age of 3 years. In the present study, the clinical signs were initially shown at an age between 10 months and 7 years. This is in concordance with studies of systemic rheumatic diseases in both dogs and humans [2,26]. Of the human patients studied, 65% showed a disease onset between the ages of 16 and 55, 20% suffered an onset before the age of 16 and 15% had an onset after the age of 55 [26]. The clinical signs, with stiffness most prominent after resting and joint pain that is waxing and waning and affecting different legs and joints, indicate a rheumatic disease with polyarthritis. In order to confirm a diagnosis of polyarthritis, joint fluid analysis would have been preferable. Unfortunately, this was not performed in this study, mainly because of the reluctance of the dog owners. This procedure involves sedation/anaesthesia being performed on the dogs and there is always a risk of infections.

Eleven Tollers with signs of disease were radiographically examined with no radiographic findings. It is important to exclude other causes of joint pain, especially in older dogs. Two out of three of the oldest dogs in the study were among the dogs that were radiographically examined (with no radiographic findings). None of the dogs included in the study showed any joint swelling. Joint involvement in SLE and in some other ANA-positive rheumatic diseases is classically described as non-erosive, non-deforming arthritis with the absence of erosions on radiographs [11,27].

Many of the systemic autoimmune diseases, in both dogs and humans, present circulating ANAs. Such antinuclear antibodies can be demonstrated by the indirect immunofluorescence (IIF) ANA test. The IIF-ANA test is evaluated by fluorescence microscopy and the importance of substrate has become apparent [21,28,29]. Earlier investigations have shown that the kind of substrate used when conducting the IIF-ANA test is of the utmost importance to avoid false positive results. In our experience, the use of the monolayer of human epithelial-2, HEp-2, cells, has provided a reliable tool to avoid such spurious results in apparently healthy individuals or in patients with other inflammatory disorders [19]. However, supportive clinical signs are always required to establish diagnostic features for systemic autoimmune diseases [2].

Seventy per cent of the Tollers with signs of disease displayed a positive IIF-ANA test, with 61% of these dogs showing a speckled IIF-ANA fluorescence pattern and 39% a homogeneous staining pattern. All the sera displaying a homogeneous IIF-ANA staining pattern concomitantly displayed reactivity towards chromosomal regions in mitotic cells. The association between a homogeneous staining pattern and a concomitant chromosomal reactivity has previously been shown both for human and canine patients [2,19,21].

In an earlier study [2], canine patients with IIF-ANA-positive systemic autoimmune disease could be subdivided into 2 groups according to clinical signs and the IIF-ANA pattern. The patients with a homogeneous IIF-ANA staining pattern, and a concomitant chromosomal staining, had a tendency to show clinical signs compatible with systemic lupus erythematosus, SLE (with e.g. musculoskeletal disorders, skin disorders, anaemia, thrombocytopenia, polymyositis and fever). An SLE-related disorder was the probable diagnosis of the dogs displaying speckled IIF-ANA staining patterns. The SLE-related diseases may, as in human patients, have overlapping clinical features and may show ANA reactivity towards different (usually non-chromosomal) nuclear antigens [2,14,21]. There is, of course, a possibility that the Toller breed is affected by different immune-mediated diseases (including SLE or SLE-related disorders), giving rise to the different IIF-ANA appearances. Another explanation could be that the disorder of the Tollers indicates a common immunological and/or genetic disturbance, giving rise to different kinds of autoantibody production.

The Tollers in this study thus display different IIF-ANA reactivity, although they show very similar clinical signs. Although other concomitant signs of disease were unusual, 6 dogs displayed skin changes. When comparing signs such as stiffness, joint pain, muscle pain, skin changes and fever with different IIF-ANA reactivity, there was no clear-cut association found. Among the Tollers showing the different clinical signs, the ANA positivity varied from 67–75%, i.e. close to the overall picture of a 70% ANA positivity among the sick Tollers included in the study. One observation, however, was that 4 out of the 6 dogs with skin changes displayed homogeneous IIF-ANA fluorescence patterns. Skin changes have been reported as part of several systemic rheumatic disorders in both humans and dogs, especially in those cases with a probable SLE diagnosis [2,6,9,11,27]. One of the Tollers with a homogeneous IIF-ANA pattern showed vitiligo and another dog showed plasmacytic and pyogranulomatous dermatitis, both of these disorders with a probable immune-mediated origin [30,31]. However, the other four dogs with skin problems displayed unspecific lesions whose background is unclear.

The possible systemic rheumatic disorder in the Tollers seems to be associated with a familial predisposition, as five of the dogs with signs of disease came from the same litter and another dog with disease had the same father as the litter-mates. This is in concordance with the study investigating Toller patients with another immune-mediated disease, SRMA, where it was strongly indicated that genetic factors are involved in the aetiology of the disease [3]. The six dogs mentioned above all entered the study at 1–2 years of age, showed similar clinical signs and displayed the same IIF-ANA staining pattern. However, two other closely-related dogs (father and daughter) displayed different IIF-ANA staining patterns.

The Tollers showing clinical signs of disease displayed higher fibrinogen and alpha-2 globulin values and lowered numbers of lymphocytes in comparison with the control dogs. The increased fibrinogen and alpha-2 globulins may be caused by the on-going inflammatory reaction. However, the mean values for these parameters were within the reference intervals, and therefore the changes probably are of minor proportions. Lymphopenia and increased acute phase proteins may also be seen in connection with treatment with corticosteroids. There were only five Tollers that were receiving treatment with corticosteroids when the blood samples were taken. Even if these five dogs on glucocorticoid treatment were excluded, the diseased Tollers still had significantly higher fibrinogen values and lowered lymphocyte counts.

Glomerulonephritis is reported both in man and dogs to be a common finding in certain systemic autoimmune diseases, due to immune complex deposition in the glomeruli [6,12]. In this study, urine analysis was performed for 17 diseased dogs and none of them showed proteinuria, isosthenuria or any other sign of kidney disorder. However, in one of the dogs for which a urine sample was not available, the serum albumin value was very low (9 g/L). Unfortunately, it was not possible to continue to perform further examinations on this dog and it died 3 months after being put on corticosteroid treatment. The low serum albumin value in this case may be indicative of a kidney disorder, in spite of normal serum creatinine values.

Three of the sera from dogs with signs of disease and five samples from healthy control dogs displayed a positive titre for *Anaplasma phagocytophilum*, while one dog with signs of disease and five control dogs were positive for *Borrelia burgdorferi*. None of these dogs were suspected of suffering from a tick-borne disease, although some of the Tollers with signs of rheumatic disease were treated with antibiotics to exclude this possibility. However, the signs were not improved during this treatment. An earlier investigation [32] was performed in Sweden measuring sero-prevalence for *Anaplasma phagocytophilum *and *Borrelia burgdorferi *in dogs not clinically suspected of being infected with either of the two agents. This study showed that the overall sero-prevalence for *Anaplasma phagocytophilum *was 17.7% and that for *Borrelia burgdorferi *3.9%. Thus, the sero-prevalences are not higher in Tollers with signs of rheumatic disease than in the Swedish dog population in general. Among the healthy control dogs, 25% showed positive titres for either of the agents, which is a higher frequency than that reported in the study mentioned above. None of the healthy control dogs showed any signs of disease, neither when included in the study nor at the follow-up contacts, which took place between 4 months and 3 years later.

Corticosteroids were the most common treatment. The majority of the dogs (65%) showed good response to corticosteroid treatment, even if the treatment was gradually tapered to low doses. There was no association when comparing IIF-ANA results and the results of treatment.

The Nova Scotia duck tolling retriever constituted approximately 0.6% of the canine population in Sweden during the five years of the study, and the proportion of Tollers presented to the University Animal Hospital at SUAS in Uppsala was 0.86%. Of the serum samples analysed at the Clinical Pathology Laboratory of the University Animal Hospital in Uppsala during these five years, 121 samples had a positive IIF-ANA test. Of these 121 dogs, 32 were Tollers. Thus, the Toller breed constituted approximately 26% of the canine ANA-positive results, which suggests that Tollers seem to be clearly over-represented according to IIF-ANA positivity and thus indicates a breed-specific rheumatic disorder.

## Conclusion

In conclusion, the present paper describes a disorder in Nova Scotia duck tolling retrievers where the clinical picture includes prolonged stiffness, especially after rest, and pain from different joints. The majority of the dogs display ANA positivity and an improvement of signs when put on corticosteroid treatment. In those cases where joints were radiographically investigated, all the results were considered radiographically normal.

Taken together, these findings indicate that this disorder in Tollers is an immune-mediated rheumatic disease. However, the different ANA reactivity does not exclude the possibility that the breed is affected by different immune-mediated diseases or the theory that the disorder indicates a common genetic and/or immunological disturbance, giving rise to different kinds of autoantibody production. Therefore, further studies of other immunologic parameters and genetic investigations would be important tools for obtaining further information about this rheumatic disorder in Tollers.

## Competing interests

The authors declare that they have no competing interests.

## Authors' contributions

HHH conceived and designed the study, examined the patients and the control dogs, coordinated the different clinical tests including blood and urine sampling, evaluated the ANA test results and drafted and lead the work with the manuscript. IL participated in the design of the study, evaluated the haematology and serum biochemistry tests and the urine analyses, performed the statistical analysis and participated in the work with the manuscript. Both authors read and approved the final manuscript.
